# Combined use of Ilizarov external fixation and Papineau technique for septic pseudoarthrosis of the distal tibia in a patient with diabetes mellitus

**DOI:** 10.3402/dfa.v5.22841

**Published:** 2014-02-12

**Authors:** Stefanos D. Koutsostathis, Panagiotis Lepetsos, Vasilios D. Polyzois, Spyros G. Pneumaticos, George A. Macheras

**Affiliations:** 1 4th Department of Trauma & Orthopaedics, KAT Hospital, Athens, Greece; 2 3rd Orthopaedic Department, University of Athens Medical School, KAT Hospital, Athens, Greece

**Keywords:** tibia, infection, non-union, Ilizarov, external fixation, Papineau technique

## Abstract

The surgical treatment of open pilon fractures has a high complication rate especially in diabetic patients. In this article, we present a case of an infected tibial non-union after an open reduction and internal fixation in a diabetic patient, treated with Ilizarov external fixation combined with Papineau technique. Combined use of external fixation and Papineau technique can provide an alternative option for the treatment of septic pseudoarthrosis of the distal tibia.

Operative fixation of distal tibial pilon fractures can become quite challenging as the extensive soft tissue compromise associated with such injuries can lead to a higher complication rate. The choices of surgical treatment include a) open reduction and internal fixation, b) external fixation, c) closed reduction with internal fixation and minimal periosteal stripping with preservation of soft tissues. Surgical complication may include ankle stiffness, infection, delayed union, non-union, and arthritis (1, 2).

Wound infection is one of the most common complications of the surgical treatment of pilon fractures (3, 4). The reported deep infection rates with plated distal tibia range from 0 to 15%; however, this rate is three times higher in diabetic patients (4, 5). Such deep infections may not be easily controlled and can develop into an infected non-union. Development of infection may result from compromised soft tissue and bone vascularity, systemic compromise of the host, and virulent or resistant organisms. Infection control is achieved with radical osseous and soft tissue debridement, skeletal stabilization, and microbial-specific antibiotics (6, 7).

There are many techniques for the treatment of an infected non-union of the distal tibia but none has been absolutely convincing in their efficacy. Such treatment options include removal of retained hardware, intramedullary and extramedullary debridement of necrotic tissue, placement of bone grafts and antibiotic spacers, use of Papineau technique, and application of an Ilizarov external fixation in a single or multiple reconstructive stages (6, 8–13).

## Case report

**Fig. 1:**
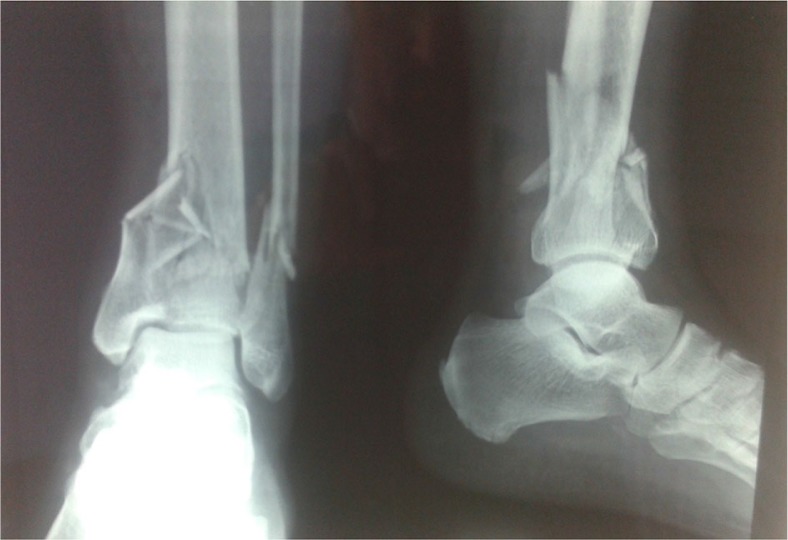
Initial lower extremity radiographs showing the distal tibial and fibular fractures.

**Fig. 2:**
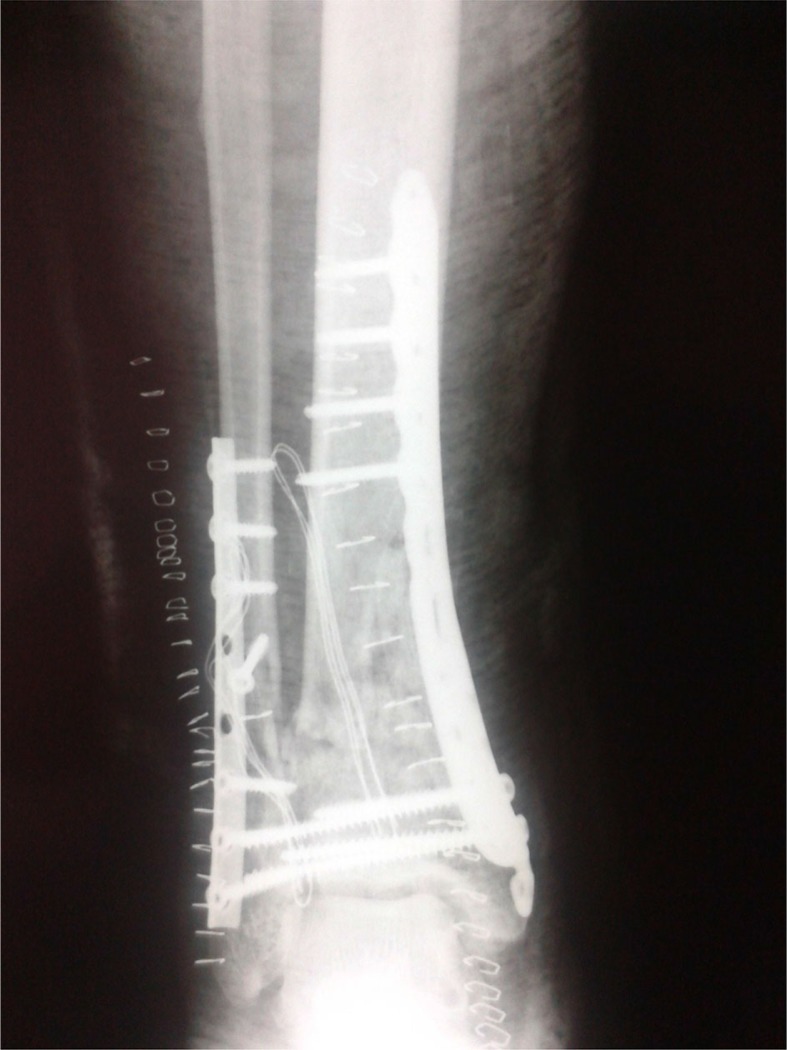
Postoperative anteroposterior lower extremity radiograph showing the internal fixation of the pilon fracture.

**Fig. 3:**
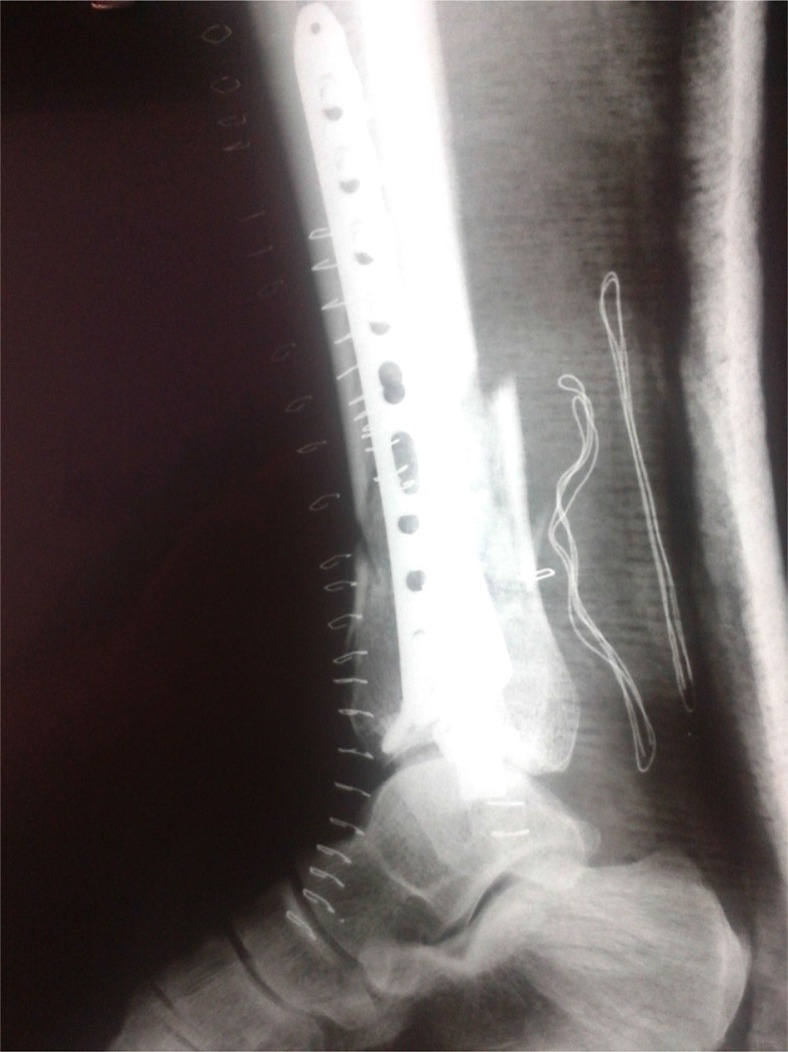
Postoperative lateral lower extremity radiograph showing the internal fixation of the pilon fracture.

A 63-year-old male presented to the emergency department of our institution in January of 2011 with an open pilon fracture of his left tibia along with a fracture of the distal fibula (Fig. 1). His medical history was significant for diabetes mellitus being treated with insulin therapy. The patient's fracture was immediately treated in the operating room with an open reduction and internal fixation with plates and screws. Postoperatively (Figs. 2 and 3), the patient received intravenous antibiotic therapy with ceftriaxone, amikacin, and metronidazole for 3 days and the patient was discharged from the hospital 1 week after the initial operation.

**Fig. 4:**
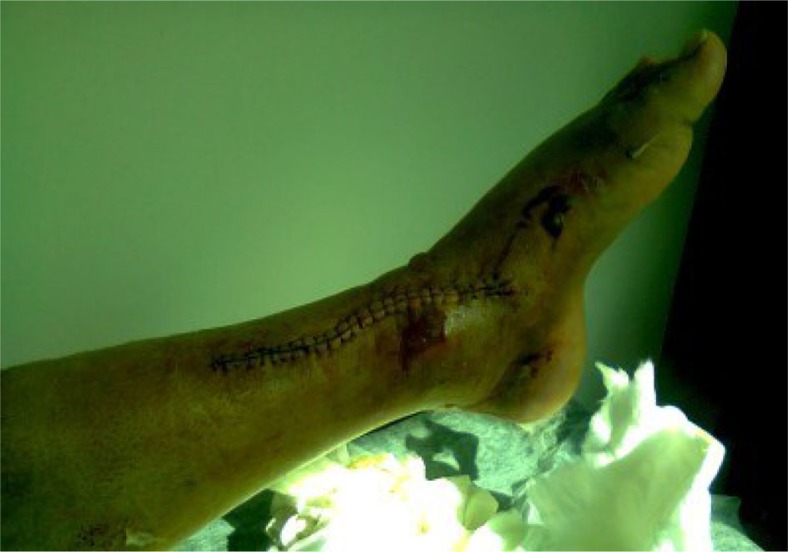
Initial presentation of the skin lesion after the internal fixation of the pilon fracture.

**Fig. 5:**
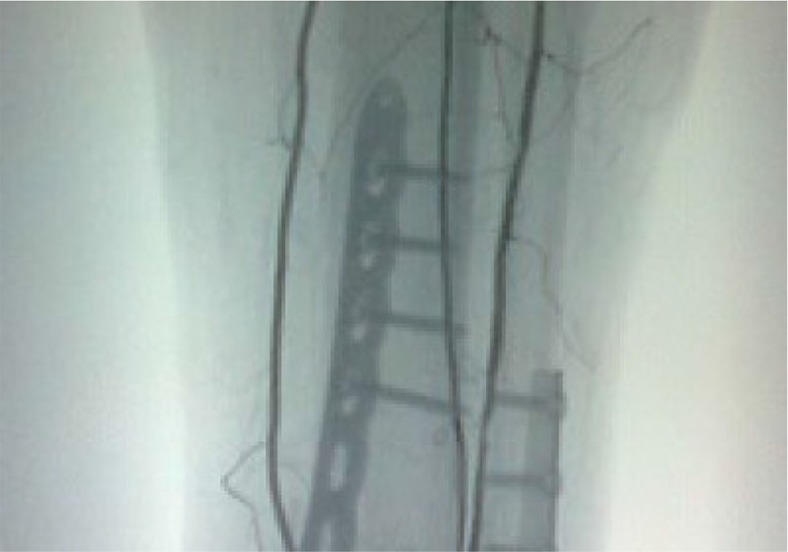
Postoperative angiography showing partial obstruction of the fibular artery.

One month after the operation, the patient presented with a skin lesion at the side of the incision (Fig. 4) with a postoperative angiography revealing a partial obstruction of the fibular artery (Fig. 5). Three months after the initial operation, the patient developed skin necrosis and infection at the site of surgical intervention. At that time, all retained hardware was surgically removed and the patient received oral antibiotic therapy for the following 3 months.

**Fig. 6:**
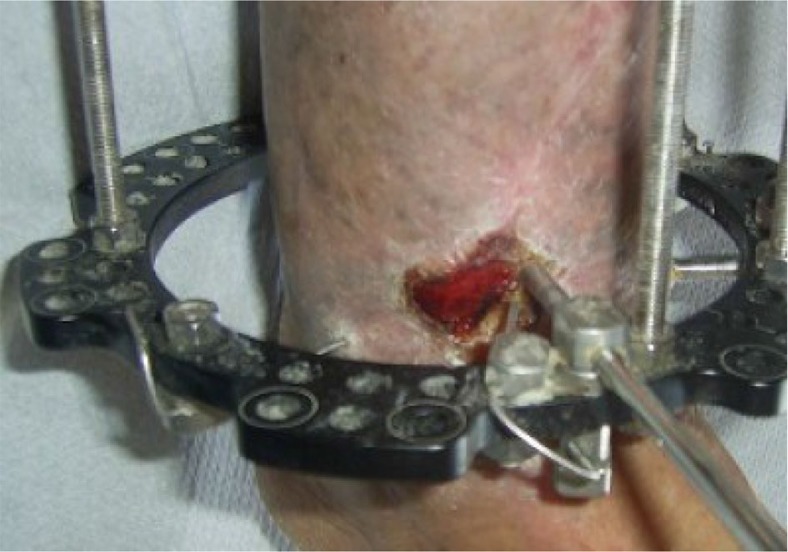
Clinical picture showing the Ilizarov external fixation combined with Papineau technique.

Six months after the first operation, the patient was diagnosed with a septic non-union of the tibia. At that time, an extensive surgical osseous and soft tissue debridement was performed in addition to skeletal stabilization and Ilizarov external fixation. The skin defects were treated with the Papineau technique (Fig. 6). Intraoperative cultures were positive for *Streptococcus mitis, Escherichia coli*, and *Stenotrophomonas maltophilia*, and biopsies were positive for inflammation. Postoperatively, the patient was allowed immediate full weight-bearing and received oral antibiotics for an additional 3 months.

**Fig. 7:**
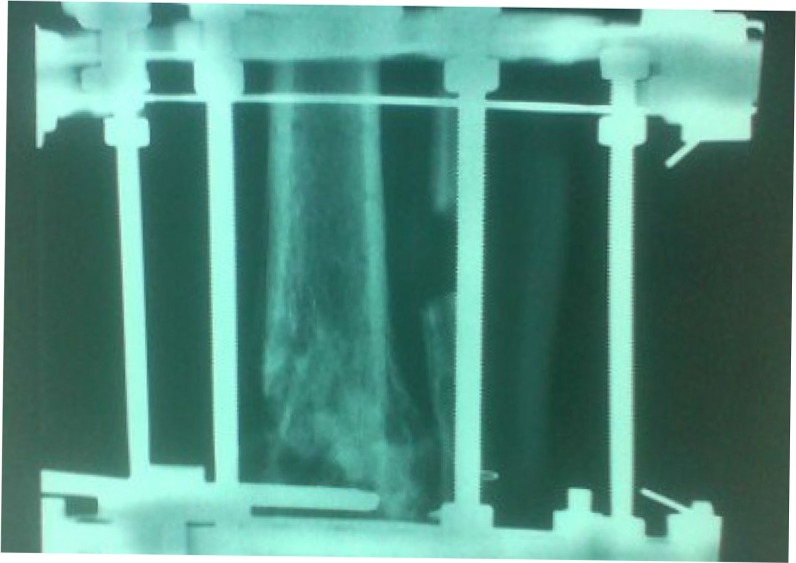
Postoperative anteroposterior lower extremity radiograph at 6 months after the Ilizarov external fixation application.

**Fig. 8:**
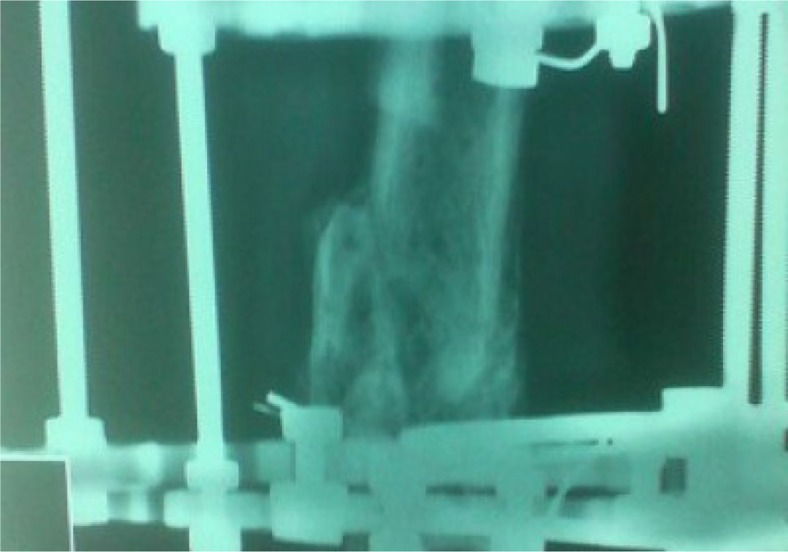
Postoperative lateral lower extremity radiograph at 6 months after the Ilizarov external fixation application.

**Fig. 9:**
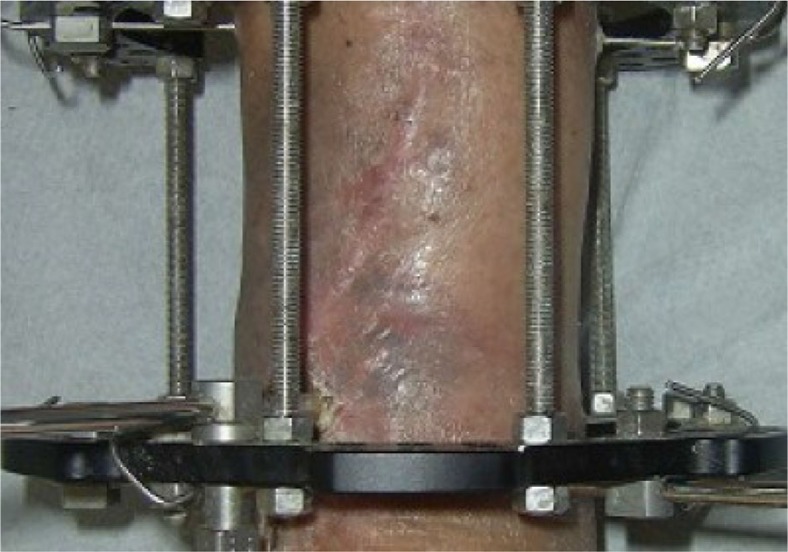
Clinical picture showing the soft tissue defect healed at 6 months after the Papineau technique.

Six months after the Ilizarov external fixation, there was radiographic evidence of fracture healing (Figs. 7 and 8). The skin defect was healed (Fig. 9) and the Ilizarov external fixation was removed. One year after the operation, the patient presented without any pain, had full range of motion at the ankle, and had returned to his daily activities.

## Discussion

Many surgical protocols have been described to treat infected non-unions of the distal tibia but few exist for the treatment of septic tibial pseudoarthrosis in the diabetic population.

In the presence of deep infections with implanted hardware for the treatment of long bone fractures, the surgeon is faced with a dilemma of initial implant preservation that may support the fracture but can certainly be the cause of continuous infection (14, 15) versus hardware removal and further stabilization with an external fixation device. With the latter treatment, the infection can normally be well controlled, but the long duration of treatment may cause marked patient discomfort and an increased possibility of pin-tract infection (16, 17). In this case report, all implanted hardware were removed and an Ilizarov external fixator was applied in staged reconstruction for complete control of the infection.

In this case report, intraoperative cultures revealed *Escherichia coli, Streptococcus mitis*, and *Stenotrophomonas maltophilia*. The presence of *Escherichia coli* has been reported to be responsible for septic tibial non-unions in previous studies (18). *Streptococcus mitis* is a commensal oral streptococcus that presents no great immunological threat but has emerged as a significant pathogen in elderly immunocompromised patients and it is implicated in a wide range of diseases (19) while *Stenotrophomonas maltophilia* is an environmental global emerging multiple-drug-resistant organism that is most commonly associated with respiratory infections in humans but can also cause osteomyelitis (20, 21).

The Ilizarov method has been used successfully for the treatment of septic tibial non-unions (22, 23). Karargyris et al. reported the use of circular external fixation in a two-stage protocol for the treatment of septic pseudoarthrosis of the tibia (24). Many studies have demonstrated the effectiveness of the Ilizarov method and circular external fixation for eradication of infected tibial non-unions (18, 25–28). Acute osseous shortening followed by distraction osteogenesis has also been reported as a successful method for the treatment of tibial non-union with bone loss (29). Takahashi et al. presented a case where fragmental bone transport in conjunction with acute shortening was followed by gradual lengthening using the Ilizarov technique for a failed infected non-union of the tibia (30).

The placement of antibiotic-loaded bone cement has also been reported for the treatment of septic pseudoarthrosis. The main advantage of this approach is the continuous local release of antibiosis over a longer period of time (31, 32). However, this technique may also present as a potential source of infection. Gulan et al. described a technique for the treatment of infected non-union of the tibia where autogenous cancellous bone from the iliac crest was placed on the anterior surface of the interosseous membrane (33). Schottle et al. reported a high rate of bone healing after two-stage reconstruction with free vascularized soft tissue transfer and conventional bone grafting within a cement-induced membrane (10). Vascularized muscle and fibular grafts have also been variably used for the reconstruction of infected tibial non-unions, but this method cannot be used in patients with extensive scarring of the lower extremity, large areas of skin loss and with questionable patency of the tibial vessels (34, 35).

Initial debridement with incomplete bone resection followed by repeated local soft tissue management was originally described by Papineau in 1973. The technique involves open cancellous bone grafting on a granulated tissue base with delayed soft-tissue coverage by skin grafting or healing by secondary intention (36). Papineau and others have reported high rates of success in eradicating chronic bone infection and addressing significant bone deficits (12, 37, 38). Saleh et al. described a modification of the Papineau technique that included the use of circular external fixation in the management of infected tibial non-unions (9). A recent study by Archdeacon et al. suggested a modification of the Papineau technique by implementing a vacuum-assisted closure device, but this method may present with challenges in patients with diabetes mellitus (39).

In conclusion, the combined use of Ilizarov external fixation and Papineau technique may be a feasible option for the treatment of infective non-union of the tibia, especially in the diabetic population. Further studies are necessary to evaluate the effectiveness of the method in a larger series of patients.
